# Circulating surfactant protein D as a potential lung-specific biomarker of health outcomes in COPD: a pilot study

**DOI:** 10.1186/1471-2466-7-13

**Published:** 2007-10-08

**Authors:** Don D Sin, Rochelle Leung, Wen Q Gan, SF Paul Man

**Affiliations:** 1 The University of British Columbia (Respiratory Division), Vancouver, BC and The James Hogg iCAPTURE Centre for Cardiovascular and Pulmonary Research (St. Paul's Hospital), 1081 Burrard Street, Vancouver, BC, Canada, V6Z 1Y6

## Abstract

**Background:**

There is a paucity of surrogate lung-specific biological markers that can be used to track disease progression and predict clinical outcomes in chronic obstructive pulmonary disease (COPD). The principal aim of this pilot study was to determine whether circulating surfactant protein D (SPD) or Clara Cell protein-16 (CC16) levels are associated with lung function or health status in patients with severe COPD.

**Methods:**

We studied 23 patients with advanced COPD. Lung function measurements, Chronic Respiratory Disease Questionnaire (CRQ) scores, and serum levels of SPD, CC16, and C-reactive protein (CRP) were determined at baseline and at 3 months.

**Results:**

At baseline, FEV_1 _was inversely associated with serum SPD levels (P = 0.045) but not with CC16 (P = 0.675) or CRP levels (P = 0.549). Over a 3 month period, changes in SPD levels correlated significantly with changes in CRQ scores (adjusted P = 0.008) such that patients who had the largest declines in serum SPD levels experienced the largest gains in health status. The association was particularly notable between circulating SPD level and the dyspnea domain of the CRQ score (P = 0.018). Changes in CC16 or CRP levels did not correlate with changes in CRQ scores.

**Conclusion:**

Changes in serum SPD levels tracked well with changes in health status over a 3 month period in patients with severe COPD. These data suggest that circulating SPD levels may be useful biomarkers to track health outcomes of COPD patients.

## Background

Over 600 million people worldwide have chronic obstructive pulmonary disease (COPD) [1]. Dissimilar to most other major causes of mortality in the world, COPD morbidity and mortality continue to escalate at an alarming rate [1]. The World Health Organization predicts by 2020, COPD will to be the 3^rd ^leading cause of mortality (currently 4^th^) and the 5^th ^leading cause of morbidity (currently 12^th^) throughout the world [1]. Despite major advances in the understanding of COPD pathophysiology, there is a dearth of effective medications that can modify its course [2]. One major limitation of drug development has been the paucity of surrogate biological markers that can be used to track disease progression (i.e. progressive decline in lung function and increase in symptoms such as dyspnea) and predict clinical outcomes [3]. COPD is a heterogeneous disorder with multiple different (but related) phenotypes. The development of lung-based biomarkers has been impeded by a variety of technical and logistical factors including lack of standardization of sample collection, invasiveness of the procedure and poor patient tolerability [3]. Blood biomarkers are conceptually more appealing owing to the relative ease of procurement and standardization of measurements. Encouragingly, recent data suggest that certain inflammatory biomarkers (e.g. C-reactive protein, CRP) correlate with disease severity and with adverse health outcomes of patients with mild to moderate disease [4]. However, although CRP is a good predictor of cardiovascular events, it is not a particularly good marker of respiratory health outcomes or rate of decline in lung function [4]. This may not be surprising given that the predominant source of circulating CRP is the liver and not the lungs [5]. To capture respiratory health events, lung-specific biomarkers are desirable. Surfactant protein D (SPD) and Clara cell protein-16 (CC16) are two proteins that are produced predominantly in the lungs and as such may be useful in tracking disease progression and health status of COPD patients [6]. The principal aim of this pilot study was to determine whether circulating SPD and CC16 are associated with lung function and health status of patients with advanced COPD.

## Methods

In this pilot study, we used serum samples obtained in a recently completed trial of non-invasive mechanical ventilation (NIMV) in advanced COPD patients. The details of the trial are published elsewhere [7]. In brief, we recruited study participants with a clinical diagnosis of COPD and who were 40 years of age or older and had at least a 10 pack-year history of cigarette smoking; forced expiratory volume in one second (FEV_1_) to forced vital capacity (FVC) ratio of less than 70% and a post-bronchodilator FEV_1 _that was less than 70% of predicted. At each visit, patients performed spirometry using a rolling seal spirometer and according to published American Thoracic Society standards, as previously described [7]. At these visits, the patients also completed the Chronic Respiratory Disease Questionnaire (CRQ) to assess their health status. CRQ is an interviewer administered questionnaire, which asks patients to rate their health status in four domains: dyspnea, fatigue, emotional function, and mastery [8].

Venipuncture was also performed at each visit during daytime. The samples were collected in plain tubes and were allowed to clot for at least 30 minutes. They were then centrifuged at 1500 × *g *for 15 minutes at room temperature, divided into aliquots using a sterile plastic transfer pipette and frozen in -80°C conditions until use. The samples were thawed once and SPD (Biovendor, Modrice, Czech Republic), and CC16 levels (Biovendor, Modrice, Czech Republic) were determined using commercially available ELISA kits. They were measured in duplicate with a coefficient of variation of 3.9% and 1.9%, respectively. All samples from the same patient were included in the same assay and were performed in duplicate with a coefficient of variation of 3.9% and 1.9%, respectively. The analytical limit of detection for SPD was 0.2 ng/mL, with a sensitivity of 1.2 ng/mL and that for CC-16 was 20 pg/ml with an assay sensitivity of 500 pg/ml. Samples were diluted 1:6 and all fell within the assay's detection range. For benchmarking, in these samples, we also measured CRP levels using a highly sensitive commercially-available solid-phase sandwich enzyme-linked immunosorbent assay kits (Alpha Diagnostics, San Antonio, Tx).

### Statistical analysis

There were 13 participants in NIMV group and 10 participants in control group. Two groups were similar in lung function, SPD, CC16, and CRP levels at baseline and after 3 months of therapy [7]. Therefore, we combined the two groups together for analytical purposes. As SPD, CC16, and CRP were non-normally distributed, we log-transformed these values to achieve normality. We then employed linear regression techniques to determine whether FEV_1 _or CRQ scores were associated with levels of these biomarkers in the systemic circulation and whether these biomarker levels were related to each other. We performed these analyses for the cross-sectional data at baseline as well as for the longitudinal data over 3 months of follow-up. For the longitudinal component of the study, we regressed the changes in the biomarker levels against changes in FEV_1 _or CRQ scores. To control for potential confounders, we conducted a stepwise regression analysis in which covariates (age, sex, body mass index, PO_2_, PCO_2 _and FEV_1_) were added to the model one by one and retained if the p-value was 0.15 or less in the multivariate analysis. All tests were two-tailed in nature and were performed using SAS software (version 9.1; SAS Institute; Cary, NC). Continuous variables are expressed as mean ± SD unless otherwise specified.

## Results

There were 23 participants in the study: 11 men and 12 women. The average age was 65 years. All of the participants had advanced COPD with an average FEV_1 _of 31% of predicted. At baseline, the median level of circulating SPD, CC16, and CRP was 74.7 ng/mL, 4.6 μg/L, and 3.5 mg/L, respectively. The baseline demographic and clinical characteristics are summarized in Table 1. The relationship between SPD and these clinical characteristics at baseline are summarized in Table 2.

**Figure 1:**
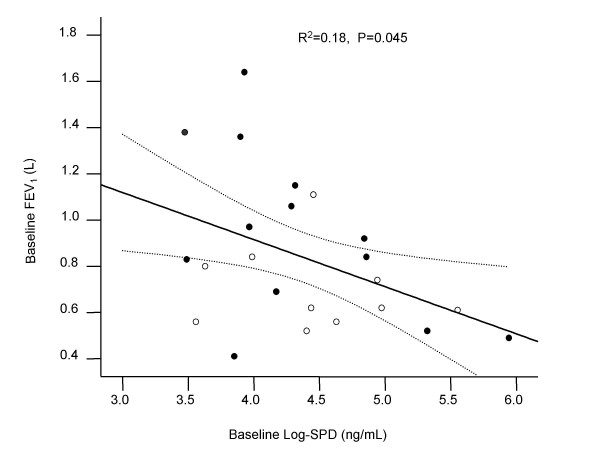
**The relationship between baseline serum Log-SPD levels and baseline FEV_1_**. Black dot, NIMV group; White dot, Control group. Abbreviation: FEV_1_- forced expiratory volume in one second, SPD – surfactant protein D

**Figure 2:**
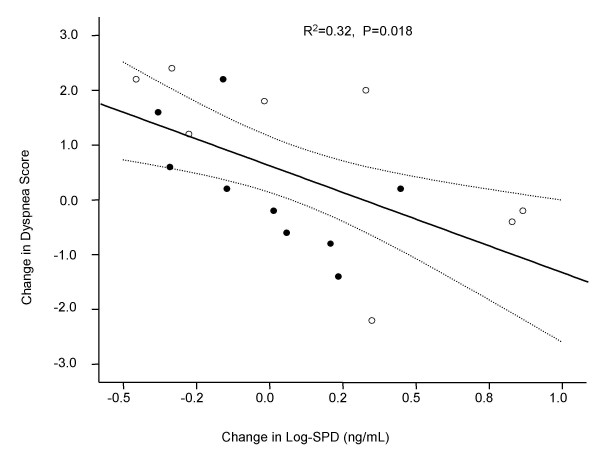
**Relationship between the Changes in serum Log-SPD levels and changes in the dyspnea Score over three months**. Black dot, NIMV group; White dot, Control group. Abbreviation: SPD – surfactant protein D

At baseline, FEV_1 _was inversely associated with SPD levels (P = 0.045) (Table 3, Figure 1) but not with CC16 (P = 0.675) or CRP levels (P = 0.549) (Table 3). There was no significant correlation between the three biomarkers at baseline (Table 3). After 3 months of follow-up, the changes in circulating SPD levels were significantly and inversely associated with the changes in CRQ score (P = 0.010, adjusted P = 0.008) (Table 4). The association was the strongest between circulating SPD levels and the dyspnea domain score of CRQ. As circulating SPD levels increased, patients experienced increase in dyspnea (P = 0.018) and worse health status (Table 3, Figure 2). CRQ scores were not associated with changes in CC16 or CRP levels (Table 4). Moreover, there was no significant overlap in the changes of these biomarkers over time (Table 4). The results from the stepwise multivariate analysis were similar to those obtained in the univariate analysis.

**Table 1:** Baseline characteristics of the study participants

	**Mean ± SD**
Number	23
Age (year)	64.8 ± 10.6
Men (%)	11 (47.8%)
Body mass index (kg/m^2^)	27.7 ± 6.6
FEV_1 _(L)	0.84 ± 0.32
FEV_1 _(% predicted)	30.8 ± 14.8
PaO_2 _(mmHg)	58.6 ± 10.0
PaCO_2 _(mmHg)	44.5 ± 9.8
CRQ score	4.1 ± 1.1
Dyspnea	3.0 ± 1.0
Fatigue	3.5 ± 1.3
Emotion	4.9 ± 1.3
Mastery	4.5 ± 1.4
Six-minute walk test (m)	286.0 ± 123.6
SPD (ng/mL)*	74.7 (49.2–128.7)
CC16 (μg/L)*	4.6 (3.8–8.7)
CRP (mg/L)*	3.5 (1.1–9.8)

* Median and interquartile range.

Abbreviations: CC16 – Clara cell protein, CRP – C-reactive protein, SPD – Lung surfactant protein D, FEV_1 _– forced expiratory volume in one second.

**Table 2:** The Relationship Between Circulating Surfactant Protein (SPD) Levelsand Clinical Characteristics

	β-coefficient*	**R-value**	**P value**
Age (year)	-0.014 ± 0.013	0.23	0.296
Male vs. female	0.138 ± 0.284	0.11	0.633
Body mass index (kg/m^2^)	-0.005 ± 0.022	0.05	0.822
PaO_2 _(mmHg)	-0.007 ± 0.015	0.11	0.629
PaCO_2 _(mmHg)	0.015 ± 0.0146	0.22	0.304
CRQ Score	-0.089 ± 0.136	0.14	0.519
Dyspnea	-0.225 ± 0.142	0.33	0.128
Fatigue	-0.022 ± 0.109	0.04	0.844
Emotion	0.007 ± 0.114	0.01	0.950
Mastery	-0.110 ± 0.101	0.23	0.288
Six-minute walk test (m)	-0.001 ± 0.001	0.11	0.629

* Regression coefficient ± SE.

Abbreviations : CRQ – Chronic Respiratory Disease Questionnaire, SPD – surfactant protein D.

**Table 3:** The Relationship Between Lung function and Serum Protein Biomarkers at baseline

	Baseline SPD*			Baseline CC16*			Baseline CRP*		
			
	**β**	**R**	**P value**	**β**	**R**	**P value**	**β**	**R**	**P value**
Baseline FEV_1_	-0.204	0.42	0.045	-0.057	0.09	0.675	0.031	0.13	0.549
Baseline SPD*		--	--	-0.293	0.23	0.295	0.080	0.17	0.446
Baseline CC16*		--	--		--	--	-0.059	0.16	0.473

* Logarithmic scale.

A negative β-coefficient indicates an inverse relationship; whereas a positive β-coefficient indicates a positive linear relationship between the two variables.

Abbreviations : CC16 – Clara cell protein-16, CRP – C-reactive protein, FEV_1 _– forced expiratory volume in one second, SPD – surfactant protein D.

**Table 4:** Association between the changes in serum protein biomarkers, lung function, dyspnea score and total CRQ scores over 3 months

	Change in SPD*			Change in CC16*			Change in CRP*			**Change in distance**^†^		
	**β**	**R**	**P**	**β**	**R**	**P**	**β**	**R**	**P**	**β**	**R**	**P**

Change in Dyspnea Score	-1.95 (-1.95)	0.56 (0.56)	0.018 (0.018)	-1.33 (-1.55)	0.24 (0.28)	0.359 (0.185)	0.42 (0.19)	0.23 (0.10)	0.367 (0.626)	-14.63 (-16.91)	0.46 (0.52)	0.063 (0.032)
Change in CRQ Score	-1.50 (-1.13)	0.61 (0.44)	0.010 (0.008)	-1.52 (-2.01)	0.38 (0.49)	0.135 (0.011)	-0.14 (-0.35)	0.10 (0.26)	0.688 (0.214)	-15.67 (-15.67)	0.36 (0.36)	0.163 (0.163)
Change in SPD*		--	--	0.64 (0.64)	0.39 (0.39)	0.119 (0.119)	0.15 (0.15)	0.29 (0.08)	0.261 (0.261)	3.05 (3.05)	0.03 (0.03)	0.916 (0.916)
Change in CC16*		--	--		--	--	0.10 (0.10)	0.30 (0.30)	0.248 (0.182)	61.38 (88.51)	0.35 (0.46)	0.175 (0.611)
Change in CRP*										-5.81 (-5.81)	0.10 (0.10)	0.698 (0.698)

* Logarithmic scale.

† Change in the distance of six-minute walk test (m).

Values in brackets were derived from a stepwise regression model in which age, sex, body mass index, PO_2_, PCO_2 _and FEV_1 _were included in the model.

Abbreviations: CC16 – Clara cell protein 16, CRP – C-reactive protein, CRQ – Chronic Respiratory Disease Questionnaire, SPD – surfactant protein D.

## Discussion

COPD poses a major health burden worldwide, accounting for nearly 3 million deaths annually [1]. The development of novel therapeutics for COPD has been impeded by lack of good surrogate markers that can track disease progression and predict clinical outcomes [3]. Biomarkers derived from exhaled condensates, induced sputums and bronchial fluids, while specific, have major shortcomings including lack of standardization and difficulty in procurement of samples that limit their application in large clinical trials or studies. Blood biomarkers are more attractive because there are well established procedures for procurement and processing of samples and standardization of measurements. In COPD, the most widely studied blood biomarker has been CRP. Although it performs well in large cohorts, clinical application is limited owing to the poor specificity of the measurement. Any inflammatory or infectious insults whether or not they are linked with COPD can modify CRP levels over time [9]. In the present study, we evaluated two lung-specific blood biomarkers as potential surrogates of health outcomes in patients with severe COPD. We found that circulating SPD was significantly associated with baseline lung function and changed with changing health status of COPD patients over a 3 month period.

SPD is a large multimeric collagenous glycoprotein weighing ~43 kDa (half of the size of albumin) and is part of the collectin family of proteins [10]. It is produced mainly by type II pneumocytes in the lungs though other cells such as pulmonary Clara cells, endothelial cells and glandular cells in the gastrointestinal tract can produce small amounts of SPD [11]. SPD plays an important role in innate immunity and in host defense responses against inhaled micro-organisms and allergens [11]. Additionally, SPD has a major function in regulating surfactant homeostasis in the lungs by modulating surfactant ultra-structure and promoting reuptake of surfactant by type II pneumocytes [12]. In general, under-expression of SPD in the lung is associated with an increased risk of infections [13], while local over-expression has been associated with chronic inflammatory conditions such as asthma [14] and interstitial pulmonary fibrosis [15]. The impact of circulating SPD on lung function is not entirely clear. In the present study, we found that at baseline FEV_1 _was inversely correlated with log-SPD concentrations in the serum such that the highest FEV_1 _values were observed in patients with SPD levels less than 90 ng/ml. Similar findings have been noted by Al-Salmi and colleagues [16] in a group of pediatric patients with interstitial lung disease, and by Krane and Griese [17] in a group of patients with cystic fibrosis. Bronchial hyperresponsiveness may also relate to increased SPD levels in serum or plasma. In a group of asthmatics, Koopman and colleagues found that serum SPD was associated with acute declines in FEV_1 _following allergen exposure [18]. Additionally, elevated circulating SPD levels may predict poor clinical outcomes. Among patients with acute respiratory distress syndrome requiring mechanical ventilation, higher plasma SPD levels are associated with a greater risk of multi-organ failure, ventilator-dependence and mortality [19]. In the present study, we found that increases in SPD were associated with reduced health status and in particular with an increase in dyspnea in patients with advanced COPD. Collectively, these data suggest that elevated serum SPD is a good marker of reduced lung function, worsening health status (especially dyspnea) and other poor outcomes in patients with lung disease. Thus, serum SPD is a promising biomarker for tracking disease progression and predicting clinical outcomes in COPD.

There were limitations to this study. Firstly, although the study subjects were well characterized, the sample size was small, increasing the risk for type I errors (i.e. false negative associations). Additionally, we performed multiple statistical comparisons, which increased the probability for type 2 errors (i.e. false positives). Larger studies will be needed in the future to validate these initial findings. Secondly, nearly all of the patients in the study had severe or very severe COPD. Thus, these data cannot be generalized to patients with mild or moderate disease. Thirdly, we did not measure biomarkers other than CRP, CC16 and SPD. Thus, the use of other lung-specific molecules as potential biomarkers in COPD is not certain. Fourthly, we did not have any broncho-alveolar samples for measurement and as such it is uncertain why FEV_1 _was inversely related to serum SPD levels. Reduced lung expression of SPD is associated with lung injury and poor lung function [20]. In general, lung-based SPD measurements correlate positively with FEV_1_, while blood-based SPD measurements correlate negatively with FEV_1_. Since lung is the major source of SPD production, this observation suggests that with lung injury, SPD and other similar proteins may leak out from the lung compartments into the systemic circulation [21], causing a paradoxical rise in serum SPD, even in the presence of decreased lung production, a notion that is supported by animal experiments [22]. There were several other notable observations in our study. Firstly, although the baseline FEV_1 _correlated with SPD, FEV_1 _failed to change as a function of SPD over 3 months. One possibility is that these two variables may not be causally related. Alternatively and more likely is that the 3 month follow-up may have been too short to observe any significant changes in FEV_1_. A larger and longer duration study is needed to confirm these preliminary observations. Secondly, although the changes in SPD related to changes in the CRQ scores, especially with the dyspnea scores over 3 months, the baseline CRQ scores were not significantly associated with baseline SPD scores. One potential explanation for this apparent paradox was the relative small spread of CRQ scores of study participants at baseline. Virtually all patients complained of poor health status (mean CRQ score of 4.1 with a SD of 1.1), which made it statistically difficult to detect any significant relationships with CRQ. A larger study with a wider variation of CRQ scores among study participants is needed to confirm these relationships.

## Conclusion

In summary, the present study suggests that in advanced COPD, serum SPD level is negatively associated with FEV_1_, and its increase over a 3-month period is associated with worsening of health status of patients. Serum SPD is a promising lung-specific biomarker to track clinical health outcomes of patients with COPD, pending validation of these early findings with larger clinical studies.

CC16: Clara Cell protein-16

COPD: Chronic Obstructive Pulmonary Disease

CRP: C-reactive protein

FEV_1_: forced expiratory volume in one second

FVC: forced vital capacity

NIMV: non-invasive mechanical ventilation

SPD: surfactant protein D

## Competing interests

The work is supported in part by the Canadian Institutes of Health Research. Don Sin is a Senior Scholar with the Michael Smith Foundation for Health Research and a Canadian Research Chair in COPD.

## Authors' contributions

All authors have made substantial intellectual contribution to the interpretation of the results and drafting of the manuscript.

## Pre-publication history

The pre-publication history for this paper can be accessed here:


